# Advertising in Medical College Announcements and in Newspapers

**Published:** 1880-12

**Authors:** 


### CORRESPONDENCE.

### ADVERTISING IN MEDICAL COLLEGE ANNOUNCEMENTS AND IN NEWSPAPERS.

1st. As long as advertising by physicians is held to be violative of the Ethical code and unbecoming the character and dignity of the medical profession, it results as a matter of certainty that *any thing* more than the mere mention of the regular branch which a professor is expected to teach during a session or course of lectures, must fall immediately under such prohibition; and consequently to expose the person acting in such a manner, to all the pains and penalties the code has enacted for the punishment of advertising physicians. It is much to be regretted that in too many instances accident, intrigue or subserviency has brought the *third* or *fourth-rate man* from his obscurity, to a place in the list of "professors" in some of the more recent medical schools, and he is not usually contented to have his name appear in these self elected faculties as professor of Anatomy, Practice, etc., alone, which it is well known cannot be exhausted by the most gifted and erudite teacher in a session of five months even while lecturing as often as is done in our best and most reputable schools. But those newly fledged "professors" attach such additional *professorial* duties to *their* names, as those of "Throat, Lungs and Heart" to a professorship of principles and practice of medicine. Or, professor of diseases of the Nervous System, and professor of Clinical Medicine as though the lungs, heart and throat should not be included in teaching practice, or that those of the nervous system did not come under a "professorship" of Clinical Medicine! If such printed appendages are not *intended* as advertisements, it may be reasonably inquired upon "what kind of meat doth *such*" professors feed that they should grow so much greater and more competent to teach, than, shall we add, wiser men? But if such as "*these* be thy gods, O Israel," then is a mighty profession fallen indeed.

These pseudo, or accidental professors are sometimes spoken of by the laity, ignorant of the methods by which their appendages are obtained, as men of some consideration, as practitioners, as they are advertised as "professor," "dean," etc, in a medical school. All such parties, too, take special pains to keep their feeble light before a credulous and gullible public, in, and out of season. Is it at all surprising or to be wondered at, that the profession is now filled with ignorant and incompetent persons, calling themselves doctors, when the *source* from which *they* obtain authority to go forth upon their mission is incompetent, corrupt, or both? "A good tree cannot bring forth evil fruit; neither can a corrupt tree bring forth good fruit." "*The tree is known by its fruit.*"

2d. ADVERTISING SURGICAL OPERATIONS *in Newspapers* is a scarcely less reprehensible practice. Furnishing secular newspapers with accounts, or reports of surgical operations performed, or cures effected by a particular process or proceeding is certainly in violation of the code of ethics, and yet these things are done by men who are members of reputable medical societies! Need we then be surprised that, with such examples before them the less prominent young beginner should seek these media for letting the public know of *his* existence and *achievements*?

My attention has been called to both these subjects, by intelligent physicians, who seeing the unblushing arrogance of the characters referred to above, and feeling themselves powerless to counteract or abate them; have requested me to aid you in holding up such abominable conduct to the observation of the profession generally. I do not hesitate to comply with the request of my friends in an initiatory communication, but would suggest, as the pages of your Journal have been declared open to every respectable physician who desires to assist in supporting the character and dignity of the profession, that others should also avail themselves of them as often as they shall find it necessary to arraign or expose the unprofessional conduct alluded to above. The time has undoubtedly come for active and persistent effort against such persons, and it is earnestly hoped that reputable medical gentlemen will engage zealously in the exposure of such conduct whenever it occurs; and continue to "strike until the last treacherous foe expires;" or is *driven in disgrace* fiom the ranks of a noble calling.

MORE ANON, M. D.

